# Entrepreneurs’ role overload and empowering leadership: A reciprocal relationship based on conservation of resources

**DOI:** 10.3389/fpsyg.2023.1118099

**Published:** 2023-03-06

**Authors:** Wei Wang, Xiaorui Zhao, Xiaomeng Zhang, Yanbin Liu, Ping Yuan

**Affiliations:** ^1^School of Business Administration, Henan University of Economics and Law, Zhengzhou, Henan, China; ^2^School of Business, NingboTech University, Ningbo, China; ^3^Economics Experimental Lab, Nanjing Audit University, Nanjing, Jiangsu, China; ^4^Logistics and E-Commerce College, Zhejiang Wanli University, Ningbo, Zhejiang, China

**Keywords:** role overload, empowering leadership, top management team heterogeneity, conservation of resources theory, role stress

## Abstract

**Introduction:**

Role overload is not new, but its increasing prevalence in recent years calls for further research. This study considers empowering leadership as a means of resource investment and proposes that it is exerted by entrepreneurs to reduce their role overload. This study adds clarity by revealing how entrepreneurs’ role overload is mitigated *via* the intermediate mechanism of empowering leadership. Hypotheses are derived from conservation of resources theory.

**Methods:**

Data were collected from 315 entrepreneurs in China using a three-round questionnaire survey.

**Results:**

This study finds that entrepreneurs’ previous experience of role overload positively relates to their current empowering leadership behavior and their previous empowering leadership behavior negatively relates to their current role overload, which implies a mediating role of empowering leadership. Specifically, the second stage of the indirect effect of previous role overload on current role overload through empowering leadership is moderated by top management team (TMT) heterogeneity. When TMT heterogeneity is higher, the negative indirect effect is stronger.

**Discussion:**

This study contributes to the idea of positive psychology and extends the scope of conservation of resources theory into the fields of entrepreneurship and leadership.

## Introduction

Entrepreneurs have to recognize and seize opportunities in the face of changing and complex business environments to gain competitive advantage (Mathias and Williams, 2017). In this process, entrepreneurs not only take on the role of founder (Dobrev and Barnett, 2005) but also the role of investor (Alvarez and Barney, 2005) and manager (Willard et al., 1992), as enterprises develop and tasks change. Different roles have different expectations of and requirements for entrepreneurs (Cardon et al., 2009; Mathias and Williams, 2017). Therefore, entrepreneurs are required to bear diverse responsibilities with multiple skills, which may lead to role overload. Role overload describes situations where entrepreneurs feel that there are too many responsibilities or activities expected of them given the time available, their abilities, and other constraints (Rizzo et al., 1970). Empirical evidence indicates that role overload is one of the most frequent stressors faced by entrepreneurs (Harris et al., 1999; Wincent and Örtqvist, 2009; Stroe et al., 2018). Previous research has indicated that role overload has multiple negative consequences and has explored entrepreneurs’ potential attitudinal and behavioral reactions toward it (Stroe et al., 2018). However, relevant evidence tends to emphasize the negative aspects of these experiences and there is a lack of positive perspectives, which constrains theoretical and practical contributions.

In this study, we present initial empirical evidence of how entrepreneurs deal with role overload by uncovering the mechanism of entrepreneurs engaging in empowering leadership behaviors to reduce role overload and the moderation of top management team (TMT) heterogeneity. To do so, we adopt conservation of resources theory (Hobfoll, 1989, 2001), which focuses on the cause of stress and describes the motivation that drives individuals to both maintain their current resources and to pursue new resources. Following this perspective, role overload was re-defined as a psychological phenomenon where role expectations are exceeded compared with individual resources available (Gilboa et al., 2008). For entrepreneurs, role overload thus occurs when they have insufficient resources to meet the expectations and requirements of different roles (Wincent et al., 2008). According to conservation of resources theory, entrepreneurs experiencing role overload are faced with a threat of a loss of resources, or an actual net loss of resources, which results in needs for protecting resources from being lost (Halbesleben et al., 2014). This inner need motivates entrepreneurs to delegate responsibilities or activities to conserve their limited resources.

When facing with role-related stress, entrepreneurs tend to change their leadership style consciously (Sharma and Kirkman, 2015), especially because entrepreneurship and leadership are not synonymous qualities (Czarniawska-Joerges and Wolff, 1991; Bolton and Thompson, 2015). Experiencing role overload means entrepreneurs detect incapacity of their current leadership style to cope with demands of multiple roles. For the purpose of conserving resources, entrepreneurs are more likely to engage in leadership that effectively assigns responsibilities of different roles to their team members. Hence, we develop and test hypotheses regarding how entrepreneurs reduce role overload by empowering their team members instead of micro-managing alone. We posit that empowering leadership is effective approach to handling role-related stress. Specifically, when experiencing role overload, entrepreneurs are more likely to engage in empowering leadership behaviors, which may subsequently help to attenuate role overload. We collected empirical data using a multi-round survey in which entrepreneurs reported their role overload at two points, separated by 2 months. During the period between the two survey points, we asked entrepreneurs to assess their empowering leadership behaviors and the heterogeneity of their TMTs. More heterogeneous TMTs perform better when converting resources into actions (Ndofor et al., 2015), which provides entrepreneurs with more opportunities for resource investment and boosts the effect of empowering leadership. We tested the moderating effect of TMT heterogeneity on the relationship between entrepreneurs’ previous role overload and current role overload *via* empowering leadership.

By shedding light on strategies for entrepreneurs to decrease role overload, we make a threefold contribution to the literature. First, this study contributes to the idea of positive psychology by revealing the importance of entrepreneurs’ intrinsic initiative, and it confirms the viewpoint that choosing proper coping strategies (e.g., empowering leadership) is fundamental to the positive effect of role overload. Second, the study extends the scope of conservation of resources theory into the fields of entrepreneurship and leadership and provides new insights into the antecedents, consequences, and conceptualization of empowering leadership behavior. Third, the study adopts a viewpoint of resource investment in exploring the function of TMT heterogeneity and reveals that the latter is not merely a set of differences in characteristics in terms of team composition, but also a prerequisite for resource investment that can amplify the impact of empowering leadership on decreasing role overload.

## Hypotheses

### Role overload and empowering leadership

Entrepreneurship is an activity where entrepreneurs and their teams identify a business opportunity, then acquire and deploy the necessary resources required for its exploitation. In this process, entrepreneurs must act as founders, investors, and managers (Willard et al., 1992; Alvarez and Barney, 2005; Dobrev and Barnett, 2005), in face of a scarcity of resources and the burden of tasks (Rizzo et al., 1970). Entrepreneurs experience role stress when they take on these roles and the demands and expectations that go with them (Kahn, 1980; Reilly, 1982; Coverman, 1989; Beehr and Glazer, 2005).

Role overload is a critical role-related stressor for entrepreneurs, along with role conflict and role ambiguity (Örtqvist and Wincent, 2006). Entrepreneurs are universally overburdened because they must deal with heavy workloads, business risks, multiple commitments, pressure from diverse sources, and the need for achievement (Rahim, 1996; Harris et al., 1999; Stroe et al., 2018). Entrepreneurs use their time, energy, and resources to create value for others, and therefore they have to face an enormous series of potential external stressors and, as role overload manifests, an accompanying increase in the intensity of workload (Parasuraman et al., 1996; Örtqvist and Wincent, 2006). Role overload is one of the role stressors that describes the degree to which individuals are cognitively overtaxed due to time pressure, commitments, and responsibilities exceeding the available capabilities and resources (Reilly, 1982). More specifically, role overload occurs when entrepreneurs are driven to reach different or even mutually exclusive goals at the same time. Hence, entrepreneurs are more likely to experience role overload when they cannot manage a high level of demands within a limited time frame or they do not have the corresponding capacities to handle such demands (Kahn et al., 1964; Peterson et al., 1995; Wincent and Örtqvist, 2009; Stroe et al., 2018). Previous research has shown that role overload has potential impacts on individuals’ mentality, behavior, or even physiology. For example, role overload contributes to a higher level of job burnout (Vullinghs et al., 2020) and emotional exhaustion (Shantz et al., 2016), and then lower job embeddedness (Karatepe, 2013) and job satisfaction (Coverman, 1989). Role overload consumes time, energy, and resources, which weakens the engagement of OCBs (Montani and Dagenais-Desmarais, 2018) and job crafting (Solberg and Wong, 2016). Moreover, evidence also shows that role overload may lead to physical symptoms such as eye strain, sleep disturbance, and headache (Nixon et al., 2011), anxiety and frustration (Kahn et al., 1964; Macewen et al., 1992), which negatively affect individual physical well-being (Alfes et al., 2018; Coverman, 1989; Örtqvist and Wincent, 2006). In summary, previous research provides sufficient evidence showing the negative impacts of role overload, but to some degree ignores the question of how individuals suffering from role overload react to and handle it proactively.

Following conservation of resources theory, we posit that resource is the key factor that determines how entrepreneurs deal with role overload. Role overload implies that the resources possessed by entrepreneurs are inadequate to meet the requirements of different roles (Wincent et al., 2008). In this case, heavy workloads, along with excessive time pressure and responsibilities, result in the continual consumption of entrepreneurs’ resources. According to the principle of primacy of resource loss, entrepreneurs who experience a threat of resource losses or an actual net loss of resources are more likely to make attempts to protect resources from being lost (Hobfoll, 1989; Halbesleben et al., 2014). More specifically, when entrepreneurs experience a high level of role overload, psychological needs for protecting resources from being lost are generated. Due to these needs, entrepreneurs are motivated to engage in behaviors that can help them to protect against and recover from resource loss, as well as gain resources (Halbesleben et al., 2014). Hence, entrepreneurs are more likely to involve their colleagues by giving autonomy and delegating authority, leading to a process of power-sharing, which can be seen as a manifestation of empowering leadership behavior (Konczak et al., 2000; Chen et al., 2007; Lorinkova et al., 2013). Even though by engaging in empowering leadership entrepreneurs have to consume their remaining resources (e.g., power and authority), relevant behaviors such as giving autonomy and delegating authority are driven by entrepreneurs’ motivations for protecting against resource loss. With strong instrumental purposes, entrepreneurs can get payback by their empowering activities from their team members who have received corresponding resources and helped achieve goals instead (Halbesleben and Bowler, 2007). Therefore, empowering leadership behaviors act as resource investment and help entrepreneurs recover from losses and gain more resources in the future (Halbesleben et al., 2014). To sum up, we propose that entrepreneurs experiencing role overload are threatened by resource losses and are more likely to act in empowering ways to compensate for the consumption of resources that role overload brings about.

*Hypothesis 1:* Entrepreneurs’ previous role overload positively relates to their current empowering leadership behavior.

Entrepreneurs tend to engage in empowering leadership behaviors when they are faced with threats of resource loss or actual losses. In this case, such behaviors act as defensive efforts by entrepreneurs to conserve their remaining resources (Hobfoll, 1989). By giving their colleagues autonomy and sharing power, entrepreneurs are more likely to increase their capacity to use their remaining resources instead of suffering incessant role overload. Moreover, empowering leadership is conceptualized as a proactive and motivational initiative that encourages team members to assume authority and autonomy (Offermann and Hellmann, 1997; Sharma and Kirkman, 2015). Entrepreneurs exert corresponding behaviors as a means of resource investment, which accords with the second principle of conservation of resources theory (Halbesleben et al., 2014). Meanwhile, authorized team members are motivated to set their own goals and meet their work commitments with increased decision-making authority (Manz and Sims, 1987; Kirkman and Rosen, 1999; Sharma and Kirkman, 2015), which helps ease the workloads of entrepreneurs and functions as the payback for resource investment. Through empowerment, entrepreneurs can concentrate on the execution of their primary work tasks, while their colleagues can also help reduce the workload burden by delegating responsibilities (Leach et al., 2003). Consequently, we posit that practicing empowering leadership may help entrepreneurs to diminish the unfavorable aspects of role overload. More specifically, empowering leadership reveals the underlying mechanism of how entrepreneurs react toward and cope with their role overload and serves as a potential linkage of the mediating path.

*Hypothesis 2:* Entrepreneur’s previous empowering leadership behavior negatively relates to their current role overload.

*Hypothesis 3:* Empowering leadership mediates the relationship between previous and current role overload.

### The moderating role of TMT heterogeneity

TMTs lay the foundation for the success of entrepreneurial firms (Eisenhardt, 2013). A considerable body of evidence has indicated that TMT characteristics are associated with financial performance (Michel and Hambrick, 1992; Certo et al., 2006; Menz, 2012). Previous research has also suggested that diversity in the managerial backgrounds and demographic characteristics of TMTs is advantageous (Barsade et al., 2000; Carpenter, 2002). Scholars have also considered TMT heterogeneity as a key factor that influences firm performance such as strategic choices (Certo et al., 2006; Chaganti et al., 2016), and found TMT heterogeneity could improve firm innovation and growth (Boone and Hendriks, 2009). TMT heterogeneity is conceptualized as a compositional characteristic that contributes to a TMT’s cognitive and information-processing capacities (Alexiev et al., 2010). Heterogeneity refers to the extent to which TMT members are different from each other in terms of demographics, functions, and backgrounds (Simons et al., 1999; Van Knippenberg and Schippers, 2007). Heterogeneous TMTs are better at problem-solving, making judgments, and taking decisions (Hinsz et al., 1997; Van Knippenberg et al., 2004). Entrepreneurs, especially in new venture teams, have to deal with role stress from diverse sources with scarce resources (Stroe et al., 2018). TMT members with different characteristics are more likely to provide diverse expertise, and in this case, entrepreneurs are more likely to find appropriate candidates from TMTs to share responsibilities and power with. Then, it is not necessary for entrepreneurs to act as different roles simultaneously because TMT members can help take responsibilities and meet requirements of these roles. Entrepreneurs’ engagement in empowering leadership behaviors is more effective in alleviating their role overload with the help of TMT members.

Moreover, TMT members play critical roles in collecting and managing resources required by strategic actions (Thompson, 2003), as well as resource utilization by converting the firm’s resources into actions and implementations (Ndofor et al., 2011) and heterogeneous TMTs have been shown to perform better when converting resources into actions and outcomes (Ndofor et al., 2015). It is inefficient when entrepreneurs utilize limited resources all by themselves, and overload comes along (Rizzo et al., 1970). By sharing partial resources such as power and authority, entrepreneurs reduce pressure due to increases in efficiency resulting from TMT members’ access to the resources. Since empowering leadership can be taken as a means of resource investment (Halbesleben et al., 2014), TMT members receive resources from entrepreneurs and utilize the resources to assist actions and implementations. By investing a certain amount of resources through empowering leadership, entrepreneurs will avoid more resource losses or attain more resource gains in the future because heterogenous TMTs effectively use the resources and help take the workload off entrepreneurs. In this process, entrepreneurs’ experience of role overload gets eased.

Hence, we propose that TMT heterogeneity strengthens the impact of empowering leadership on role overload.

*Hypothesis 4:* TMT heterogeneity moderates the second stage of the indirect effect of previous role overload on current role overload through empowering leadership. When TMT heterogeneity is higher, the negative indirect effect is stronger.

**Figure 1:**
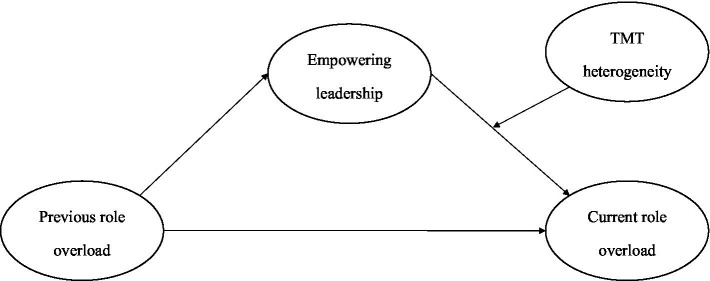
The hypothesized model.

Based on these hypotheses, we propose a new moderated mediation model that outlines the context where empowering leadership mediates the relationship between previous and current role overload and the indirect effect are likely to be influenced by TMT members’ diversity. The theoretical model is schematically represented in Figure 1.

## Methods

### Sample and procedure

We collected data from 315 entrepreneurs of 315 enterprises distributed among 56 incubators in South China. Participants’ age ranged from 22 to 52 years (Mean = 35.16, SD = 5.56), and 82.9% of them were male. Data were collected for 3 months (April 2022–June 2022), and surveys were conducted monthly. The first round of measures asked entrepreneurs to assess their role overload in early April. Then they were asked to rate themselves on empowering leadership a month later. In early June, the third-round survey asked entrepreneurs to evaluate role overload and TMT heterogeneity.

Electronic copies of questionnaires were distributed to entrepreneurs by email. In the first and second rounds, 512 and 426 responses were obtained, respectively. In the final round, valid feedback from 315 entrepreneurs was acquired. Participants were made aware of the confidential nature of their responses in our data handling and supplied with informed consent forms stating that all data collected would only be used for research purposes.

### Measure

The present study used three questionnaires. The first-round version contained demographic and control variables and items about role overload. The second-round version measured empowering leadership, and the third-round survey measured role overload and TMT heterogeneity.

All items were extracted from existing literature and adapted to fit this study. All measures were translated to Chinese following a procedure of standard translation-back-translation (Reynolds et al., 1993). All the items used Likert-type scales (1 = *strongly disagree* to 5 = *strongly agree*).

Role overload was measured with a scale based on items from Schaubroeck et al. (1989) and Beehr et al. (1976). Three items were “The amount of work I am expected to do is too great,” “I never seem to have enough time to get everything done at work,” and “It often seems like I have too much work for one person to do.” Cronbach’s alpha of the first measure and second measure were 0.96 and 0.84, respectively.

Empowering leadership was measured using a 38-item scale adapted from Arnold et al. (2000). This measure included five dimensions: leading by example, participative decision-making, coaching, informing, and showing concern/interacting with the team. Sample items were “I set high standards for performance by my own behavior,” “I listen to my work group’s ideas and suggestions,” “I help my workgroup see areas in which we need more training,” “I explain company decisions,” and “I care about work group members’ personal problems.” The fit indexes for four first-order factors plus one second-order factor fell within an acceptable range (*χ*^2^ [655, *n* = 315] = 1132.76, TLI = 0.92, CFI = 0.91, RMSEA = 0.06), indicating that these dimensions captured the distinctiveness, as well as collective reflectiveness, of the overall construct. Cronbach’s alpha was 0.93.

TMT heterogeneity was measured by a 5-item scale adapted from Lewis (2003). Sample items were “Each team member has specialized knowledge of some aspect of our project,” and “Different team members are responsible for expertise in different areas.” Cronbach’s alpha was 0.76.

### Analytic strategies

We ran a confirmatory factor analysis using AMOS 22.0 and adopted five general indices to assess the model fit: *χ*^2^/*df*, TLI, CFI, RMSEA, and SRMR (Hu and Bentler, 1999). The acceptable cut-off values that we used were <3.00 for *χ*^2^/*df*, more than 0.90 for TLI and CFI, and <0.08 for RMSEA and SRMR, which are widely reported and recommended (Hu and Bentler, 1999; Kline, 2011).

We tested the hypothesized model (a moderated direct and indirect effects model) using bootstrap methods, applying the PROCESS macro (version 4.0), which was first developed by Hayes (2013) and has been iteratively updated until 2021. According to Hayes (2015), the effect of a second-stage moderated mediation is mathematically a linear function of the moderator; and the slope of this function is a product of the coefficient of MW on Y and the coefficient of X on M,^1^ which is also called an INDEX of the moderated mediation. If this index differs from zero, it leads to the expectation that an indirect effect is moderated. We used 5,000-sample bootstrapping in this study for all the computations to yield 95% bias corrected confidence intervals. If the confidence interval excludes zero, it leads to the inference that the indirect effect is linearly related to the moderator (Hayes, 2015).

## Results

Table 1 presents a statistical summary and bivariate correlations of the variables.

**Table 1:** Descriptive statistics and intercorrelations of variables.

	Pearson correlations										
	Mean	SD	1	2	3	4	5	6	7	8	9
1 Age	35.16	5.56									
2 Gender	0.83	0.38	0.02								
3 Education	2.37	0.55	0.06	−0.03							
4 TMT size	3.80	0.67	−0.02	0.04	0.56^**^						
5 Firm size	96.33	45.55	0.03	−0.63^**^	0.10	0.12^*^					
6 Previous role overload	2.97	0.93	−0.03	0.01	−0.17^**^	−0.12^*^	0.14^*^	**0.96**			
7 Current role overload	3.20	0.75	0.01	0.02	0.13^**^	−0.03	0.11	−0.20^**^	**0.84**		
8 Empowering leadership	2.98	0.42	−0.09	0.06	−0.13^*^	0.04	−0.07	0.36^**^	−0.46^**^	**0.93**	
9 TMT heterogeneity	3.57	0.61	0.05	−0.04	−0.06	0.12^*^	0.05	0.04	−0.17^*^	0.02	**0.76**

*n* = 315. Internal consistency coefficients are reported in bold on the diagonal. Gender was recorded as male = 1 and female = 0. ^*^p < 0.05, ^**^p < 0.01.

### Confirmatory factor analysis

Using AMOS 22.0, we conducted a confirmatory factor analysis to test whether our hypothesized model captured distinct constructs. The results showed that the hypothesized 4-factor model fit the data in an acceptable way, with *χ*^2^ [98, *n* = 315] = 257.17, CFI = 0.95, TLI = 0.92, RMSEA = 0.06, and SRMR = 0.05. All the observed items loaded on their respective latent factors, and the factor loadings were all significant, with a mean of 0.76 indicating that the latent variables had accredited convergent validity. Furthermore, we compared our measurement model with two alternatives: (1) previous role overload and current role overload, specified to load on one latent factor, and the other variables loading on their respective factors, which fit worse than the hypothesized model, with Δ*χ*^2^ [3, *n* = 315] = 567.49, *p* < 0.01; (2) a 3-factor solution with TMT heterogeneity and current role overload loading on one latent factor and the other variables loading on their respective factors, which provided a worse fit than the hypothesized model, with Δ*χ*^2^ [6, *n* = 315] = 403.96, *p* < 0.01. These results indicated that the six constructs captured distinctiveness as expected in the present study.

### The mediating role of empowering leadership

Table 2 presents the result of the mediating effect. The effect of previous role overload on empowering leadership and the effect of empowering leadership on current role overload are both significantly negative, with *b* = 0.16 (*p* < 0.01) and *b* = −0.83 (*p* < 0.01), respectively, thus supporting Hypotheses 1 and 2.

**Table 2:** The regression analysis of mediating effect.

Effect	*B*	SE
Direct effect of X on M	0.16^**^	0.02
Direct effect of M on Y	−0.83^**^	0.09
Total effect of X on Y	−0.16^**^	0.05
Direct effect of X on Y	−0.03	0.04

*n* = 315. All coefficients reported are unstandardized. ^**^p < 0.01.

Furthermore, we adopted bootstrap methods to test the mediating role of empowering leadership by the SPSS PROCESS macro (version 4.0), which takes indirect effect into consideration (Shrout and Bolger, 2002). The mediating effect was tested with the expectation that the indirect effect should be non-zero (MacKinnon et al., 1995). The result shows that the indirect effect of previous role overload on current role overload *via* empowering leadership was −0.13 (95% CI [−0.1866, −0.0766]). The model fit of the mediating effect was acceptable (*R^2^* = 0.46, *F* (2, 312) = 43.01, *p* < 0.01). With the confidence interval excluding zero, thus Hypothesis 3 is supported.

### The moderating role of TMT heterogeneity

The regression results of PROCESS are shown in Table 3. Furthermore, we bootstrapped 5,000 samples and calculated the conditional indirect effects at three levels of TMT heterogeneity (−1 SD as Low, +1 SD as High, and Mean). The conditional indirect effect of previous role overload on current role overload was also computed by PROCESS, as shown in Table 4. All the confidence intervals exclude zero, indicating that the conditional effects are significant (*p* < 0.05). The INDEX of the moderated mediation model, computed by PROCESS, was −0.03 (95% CI [−0.0563, −0.0009]), and thus Hypothesis 4 is supported.

**Table 3:** Regression results of PROCESS.

Path estimated	Empowering leadership		Current role overload	
	*B*	SE	*B*	SE
Age	−0.01	0.00	0.02	0.00
Gender	0.01	0.08	0.01	0.00
Education	−0.12	0.05	0.07	0.03
TMT Size	0.11	0.04	−0.05	0.03
Firm Size	−0.00	0.00	−0.00	0.00
Previous role overload	0.16^**^	0.02	−0.01	0.02
Empowering leadership			−0.13^**^	0.17
TMT heterogeneity			−0.54^**^	0.13
Empowering leadership×TMT heterogeneity			−0.16^**^	0.05
*R*^2^_EL_	0.40^**^			
*R*^2^_CRO_			0.49^**^	

*n* = 315. Table values are path estimates from the estimated model. Entries are unstandardized coefficient estimates. EL refers to empowering leadership, and CRO refers to current role overload. ^**^p < 0.01.

**Table 4:** The conditional indirect effect.

Moderator	Effect	SE	LLCI	ULCI
Low	−0.11	0.02	−0.1589	−0.0723
Mean	−0.13	0.02	−0.1760	−0.0844
High	−0.14	0.03	−0.2018	−0.0936

**Figure 2:**
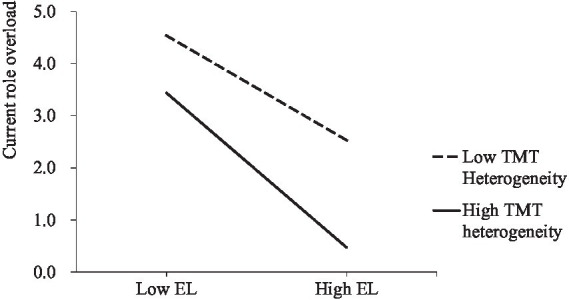
Interactive effect of empowering leadership and TMT heterogeneity on current role overload (EL refers to empowering leadership).

To visualize the moderating effect of TMT heterogeneity, we plotted the interaction of empowering leadership and TMT heterogeneity on current role overload, as shown in Figure 2. The plot indicates that when TMT heterogeneity is higher, the negative impact of empowering leadership on current role overload is stronger.

### Robustness checks

We conducted robustness checks using different operationalizations of TMT heterogeneity. Previous research suggests that team heterogeneity can be operationalized by functional background heterogeneity, educational background heterogeneity, and tenure heterogeneity (e.g., Hambrick et al., 1996). Functional background heterogeneity was measured by a variation of the Herfindal-Hirschman index,


$$H=1-\overset{16}{\underset{i=1}{\sum }}p_{i}^{2}$$

where *H* stands for the measure of heterogeneity, and *p* is the percentage of TMT members in each of the 16 functional background categories provided by Hambrick et al. (1996). Consistent with Hypothesis 4, functional background heterogeneity still negatively moderates the second stage of the mediation model, with the INDEX –0.26 (95%CI [−0.4192, −0.0978]). For educational background heterogeneity, we still used the H index and coded each member’s educational background using a set of eight different disciplines (Hambrick et al., 1996). The results of this measure of TMT heterogeneity also support Hypothesis 4, with the INDEX –0.13 (95%CI [−0.2816, −0.0469]). We calculated tenure heterogeneity using the standard deviation of the number of years the TMT members had spent in the firm. The results indicated that tenure heterogeneity negatively moderates the second stage of indirect effect, with INDEX –0.02 (95%CI [−0.0269, −0.0087]), also supporting Hypothesis 4.

## Discussion

Previous research has investigated entrepreneurs’ role overload and its management (Harris et al., 1999; Wincent and Örtqvist, 2009; Stroe et al., 2018). In this study, we explore the effects of entrepreneurs’ role overload on empowering leadership, the underlying mechanism of their reactions toward role overload, and the moderating role of TMT heterogeneity. More specifically, the results of our empirical test demonstrated that entrepreneurs’ role overload leads to empowering leadership, which in turn helps reduce role overload, and that the impact of empowering leadership on decreasing role overload is amplified by TMT heterogeneity. Our findings show that entrepreneurs experiencing role overload are motivated to engage in empowering leadership to prevent resource losses, through which they can diminish role overload. They also demonstrate that heterogeneous TMTs are beneficial to strengthening the impact of empowering leadership on role overload. These findings carry several implications for research into role theory and conservation of resources theory, as well as empowering initiatives and practice in entrepreneurship.

### Theoretical implications

This study extends our knowledge of entrepreneurs’ proactive reactions toward role overload and its underlying mechanism, making theoretical contributions in three notable ways. First, we explored entrepreneurs’ reactions toward role overload and corresponding behavioral outcomes that stem from a positive perspective, which contributes to the idea of positive psychology by revealing the importance of entrepreneurs’ intrinsic initiative. Entrepreneurship requires participants to engage in complex economic activities and entrepreneurs must use their initiative to cope with a variety of dilemmas and challenges (Frese and Gielnik, 2014). Therefore, excessive attention to the negative outcomes of entrepreneurs’ role overload risks confining the scope of inquiry and ignores the strong motivations that entrepreneurs possess to deal with numerous difficulties, including role-related stress. Following positive psychology and positive organizational behavior (Seligman and Csikszentmihalyi, 2000; Luthans, 2002a,b), our findings reveal empowering leadership as a significant outcome of entrepreneurs’ role overload. Previous evidence has indicated that role overload leads to negative consequences such as job burnout (Vullinghs et al., 2020), emotional exhaustion (Shantz et al., 2016), and job dissatisfaction (Coverman, 1989). Relevant findings imply that individuals suffering from role overload are passive recipients of role stress, rather than active agents that take the initiative to decrease its influence. This study revealed the mediating role of empowering leadership, positing a positive loop where role overload leads to empowering leadership that further weakens role overload.

Moreover, combining with post-study interviews, our findings indicate that empowering leadership is one of proper coping strategies toward role overload for entrepreneurs. We conducted interviews based on critical incidents with 13 entrepreneurs who attended the survey. The results show that role overload is common among entrepreneurs, and they cope with this role-related stress using various strategies. A general way is to change leadership style because leadership is core to management while entrepreneurs are more familiar with products, markets or technologies rather than management. Based on interviewees’ descriptions of key incidents, 4 of 13 entrepreneurs are unable to distinguish between delegation and empowering leadership, and 7 of 13 delegated first and altered to empowerment later. Delegation is similar to empowering leadership in that it encourages employees to take on decision-making authority and autonomy (Sharma and Kirkman, 2015) while empowering leadership emphasizes motivational influences that stimulate employees’ confidence and self-determination (Manz and Sims, 1987; Kirkman and Rosen, 1999). According to their feedback, delegation did help reduce role overload in the short run, but those delegators failed to achieve entrepreneurs’ expected goals. Consequently, entrepreneurs had to clear up the messy situations for them, which consumed more resources. On the contrary, empowering entrepreneurs invested more resources at first, but their expected goals were generally achieved by their team members in the long term, which effectively reduced their experiences of role overload. The results confirm the viewpoint that choosing proper coping strategies (e.g., empowering leadership) is fundamental to the positive effect of role overload, which echoes the idea of positive psychology that pressure is a driving force to improve if handling it properly (Fullan, 2010).

Second, this study deepens our understanding of the antecedents, consequences, and conceptualization of empowering leadership behavior and extends the application of conservation of resources theory to entrepreneurship and leadership research. Previous studies have revealed the antecedents of empowering leadership, such as task diversity and organizational structure (Maynard et al., 2007), contextual stressors (Spreitzer et al., 1999), and individual characteristics (Lin, 2002), as well its outcomes, such as the favorable attitudes and behaviors of subordinates (Ahearne et al., 2005; Srivastava et al., 2006; Cheong et al., 2019). This study emphasizes the rationale behind the exertion of empowering leadership behaviors and their impact on leaders who exhibit them. Exploration of its mediating role indicates that empowering leadership is the outcome of increasing role overload and the antecedent of decreasing role overload. According to the principle of primacy of resource loss (Halbesleben et al., 2014), entrepreneurs exert empowering behaviors due to their motivation to prevent the further loss of resources. Compared with defensive attitudes and actions, empowering leadership behaviors are proactive and promotional (Halbesleben et al., 2014). Corresponding with the principle of resource investment (Sharma and Kirkman, 2015), empowering leadership results in resource gain and thus reduces role overload.

As mentioned before, contrary to the view that individuals tend to choose defensive strategies for protecting resources, as indicated by conservation of resources theory (Hobfoll, 1989), our findings show that entrepreneurs may choose promotional strategies for resource investment even though they are threatened by resource loss. The results respond to the appeal for positive organizational behavior (Luthans, 2002a,b) and call for further considerations about the conceptualization of empowering leadership. It is worthwhile to clarify distinctions between empowering leadership and related leadership constructs (e.g., delegation). Considering that empowering leadership was self-reported by entrepreneurs in this study, we conducted further interviews with team members of the sample to obtain their evaluation of entrepreneurs’ empowering behaviors. Their responses show that from the point of view of team members, these kinds of leadership behavior more closely resemble delegation since such behavior is understood more as task assignment rather than real empowerment. One possible reason for this phenomenon is social desirability that occurred when entrepreneurs rated themselves on favorable behaviors, especially if entrepreneurs have insufficient knowledge about the difference between empowering leadership and delegation. Another possible reason is that distortion exists in the up-down process of empowering leadership transmission. Distortion occurs due to disturbance (Byun et al., 2020) or deficient resources (Herman and Heimovics, 2010), and implies the path from the enactment of leadership to its eventual outcomes is complex. Our findings provide a starting point for exploring the relevant issues.

Third, we investigated the impact of TMT heterogeneity based on a perspective of resource investment, revealing how it coordinates with empowerment during the frequent interactions between entrepreneurs and TMTs. Previous research has probed into the function of TMT heterogeneity by discussing its relationship with firm performance (Ancona and Caldwell, 1992; Hambrick et al., 1996). From an information processing perspective, heterogeneous teams can provide more information as TMT heterogeneity serves as a source of information, which contributes to scientific decision-making and then firm performance (Jehn et al., 1999; Harrison et al., 2002; Horwitz and Horwitz, 2007; Joshi and Roh, 2009). Meanwhile, following the perspective of social categorization, TMT heterogeneity sets team members against each other due to their dissimilarity, which damages team cooperation and lowers firm performance (Hambrick et al., 1996; Knight et al., 1999; Williams, 2016). Adopting a resource investment perspective, our study takes TMT heterogeneity as an antecedent to the process of converting resources into actions. These findings provide a new understanding of how TMTs function effectively in enterprises, in which resources are exchanged and accumulated, as part of the flow of interaction between entrepreneurs and TMT members. Specifically, TMTs share pressure, receive resource investment from entrepreneurs, achieve goals by converting resources into actions and outcomes, and pay resource gain back to entrepreneurs, which can help them mitigate role overload. Moreover, since TMT heterogeneity is critical for resource utilization, it’s worth considering whether entrepreneurs’ role-related stress results from their team compositional characteristics.

### Practical implications

The findings of this study reveal several ways in which entrepreneurial practitioners can strategically focus on leadership and team formation. First, entrepreneurs should take a more positive approach to dealing with job stressors, including role overload. Following the perspectives of positive psychology (Seligman and Csikszentmihalyi, 2000) and positive organizational behavior (Luthans, 2002a,b), how leaders in difficult circumstances react toward and handle challenges is critical for constructing a motivational climate and encouraging team members’ initiative. Admittedly, entrepreneurs may choose to cope with role overload in a passive manner, such as through temporary escape or buck-passing, and return to work after recovering from the overburden. However, this strategy is detrimental in the long term (Drnovsek et al., 2016; Mueller et al., 2017; Stroe et al., 2018). To create sustainable development, entrepreneurs need to cope with role overload using promotional strategies (i.e., empowering leadership) rather than defensive strategies (i.e., evasion and buck-passing). Our findings provide empirical evidence for the effectiveness of entrepreneurs engaging in empowering leadership behaviors to handle role overload. More specifically, recommended practices for this purpose include analyzing causes of role overload, assessing and prioritizing the most valuable resources from those available, and utilizing proactive and promotional means to protect central resources by allocating peripheral resources as investment for future resource gaining.

Second, entrepreneurs and leaders should be encouraged to recognize and understand the difference between empowering leadership and delegation, and exert empowering leadership in practice instead of simply delegating. Undoubtedly, it is easier for entrepreneurs to delegate responsibilities and duties to others rather than make use of more nuanced approaches to empowerment. But delegation is a comparatively defensive strategy that only prevents resource loss in the short term and entrepreneurs adopting this passive strategy are more likely to get stuck in a loss spiral (Demerouti et al., 2004; Guo et al., 2021). Conversely, while empowering leadership is resource-consuming in the short term, it ultimately serves as a form of investment. Entrepreneurs engaging in empowering leadership are more likely to achieve positive returns and enter into a gain spiral in the long run. Previous research has differentiated empowering leadership from delegation due to its motivational influences on employees (Sharma and Kirkman, 2015). Therefore, entrepreneurs experiencing role overload should seek a balance of empowering practices. For example, leading by example requires entrepreneurs to transmit knowledge and experiences rather than pressure; they should encourage employees to participate in decision-making rather than delegating without explanation; after delegation, entrepreneurs should spend time explaining goals and expectations to employees so that they can transform resources into actions effectively; and they can provide a motivating influence by sharing their concerns with team members.

Third, TMT heterogeneity should be taken into consideration in the team formation stage. In particular, technology-based entrepreneurs should refrain from selecting a TMT made up of technicians or engineers with similar backgrounds devoted to the pursuit of new techniques but likely to place less emphasis on business issues. In this situation, TMT members are often unable to concentrate on technological issues and become distracted by role-related pressure (Stuart and Ding, 2006; Meek and Wood, 2015; Wang et al., 2022). Meanwhile, entrepreneurs should have full knowledge of TMT members’ diverse characteristics, such as educational backgrounds, professional skills, and work experience, to make the best use of these human resources as part of an overall investment strategy. An in-depth understanding of the power of heterogeneity can help entrepreneurs assign resources and delegate authority to appropriately qualified members so that resources can be converted effectively into actions and performance (Ndofor et al., 2011, 2015). In return, team members share the stress, maximize the effectiveness of their empowerment, and decrease role overload. As for investors, they should emphasize the importance of heterogeneity when providing mentoring or coaching for entrepreneurs and pay careful attention to the role pressure that investees endure during stressful situations. Additionally, TMT heterogeneity can be taken as a positive indicator for investment decision-making.

### Limitations and future directions

This study has several limitations that should be addressed in future research. First, the relationship between role overload and empowering leadership is more complicated than the results suggest. Job stressors, including role overload, may negatively predict empowering leadership because in stressful situations leaders are less effective in maintaining interactions with their colleagues and subordinates (Staw et al., 1981; Ellis, 2006). Alternatively, job stressors may result in leaders’ empowering leadership behaviors because they can prevent resource loss by involving their employees and sharing authority and power (Sharma and Kirkman, 2015). Although our findings show that role overload is positively associated with empowering leadership, controversy still exists and several issues deserve further clarification. Future research might consider exploring the moderators of the relationship between role overload and empowering leadership. For example, internal resources such as role breadth self-efficacy, defined as “perceived capability of carrying out a broader and more proactive set of work tasks that extend beyond prescribed technical requirements” (Parker, 1998, p. 835), may serve as a personal-trait resource (Hobfoll, 2001) and help provide more motivation for investment in empowering leadership. External resources such as LMX and organizational support (Hobfoll et al., 2018; Serban et al., 2022) may also positively moderate the relationship between role overload and empowering leadership. Moreover, a non-linear relationship between role overload and empowering leadership should be considered. One possibility is that entrepreneurs are too exhausted to exert empowering leadership when they experience role overload past a certain point, exhibiting an inverted U-shape curvilinear relationship.

Second, a potential issue worth consideration is source of evaluation. In this study, empowering leadership was rated by entrepreneurs themselves and future research could instead consider comparing self-reported empowering leadership by leaders with those other-rated by employees or subordinates. In particular, according to our post-study interviews, oral evaluations of empowering leadership made by colleagues and subordinates differ from entrepreneurs’ self-assessments. Probing this notable finding may help clarify the difference between empowerment and delegation (Leana, 1986; Leana, 1987; Yukl and Fu, 1999), or even buck-passing (Mann et al., 1997; Steffel et al., 2016). Moreover, all the items measured in this study were self-reported and, despite a high level of reliability, it may still lead to the risk of common method bias (Podsakoff et al., 2003). We ran a one-factor model (see Harman, 1967) to ensure that this issue did not nullify our findings, and its poor fit (*χ*^2^ [77, *n* = 315] = 1339.74, CFI = 0.48, TLI = 0.39, RMSEA = 0.23, and SRMR = 0.23) indicates that no single factor can explain a majority of the variance.

## Data availability statement

The raw data supporting the conclusions of this article will be made available by the authors, without undue reservation.

## Ethics statement

The studies involving human participants were reviewed and approved by the Academic Committee of Henan University of Economics and Law. Written informed consent for participation was not required for this study in accordance with the national legislation and the institutional requirements.

## Author contributions

WW as the first author contributes to the idea of the model and writing. XZhao as the correspondence author contributes to the data collection and analysis. XZhang as the third author contributes to writing and revising. YL as the fourth author and PY as the fifth author contributes to writing and revising. All authors contributed to the article and approved the submitted version.

## Funding

The work was supported by National Natural Science Foundation of China (grant nos. 72203101 and 72250710170), National Social Science Foundation of China (#22CGL073), Zhejiang Province Social Science Foundation (#20NDQN321YB), and Ministry of Education of Humanities and Social Science Project of China (#22YJA630109).

## Conflict of interest

The authors declare that the research was conducted in the absence of any commercial or financial relationships that could be construed as a potential conflict of interest.

## Publisher’s note

All claims expressed in this article are solely those of the authors and do not necessarily represent those of their affiliated organizations, or those of the publisher, the editors and the reviewers. Any product that may be evaluated in this article, or claim that may be made by its manufacturer, is not guaranteed or endorsed by the publisher.

## Footnotes

^1^X/Y refers to independent/dependent variable, while M/W means mediator/moderator, respectively.
